# Nonoperative treatment of recalcitrant neuritis of the infrapatellar saphenous nerve: a case series

**DOI:** 10.1186/s13256-021-02912-4

**Published:** 2021-07-15

**Authors:** Beth Pearce

**Affiliations:** Orthopaedic Associates of St. Augustine, One Orthopaedic Pl, St. Augustine, Jacksonville, FL 32086 USA

**Keywords:** Neuritis, Infrapatellar, Infragenicular, Sartorial, Saphenous nerve, Umbilical cord, Amniotic tissue

## Abstract

**Background:**

Neuritis of the infrapatellar branch of the saphenous nerve can result from iatrogenic injury, entrapment, bursitis, or patellar dislocation. Currently, there is an unmet clinical need for treating refractory neuritis nonsurgically.

**Case presentation:**

Three patients presented with persistent anterior knee pain caused by neuritis of the infrapatellar branch of the saphenous nerve that had got excellent but only temporary relief from steroid and local anesthetic nerve block. The neuropathic pain diagnostic Douleur Neuropathique 4 questionnaire and painDETECT questionnaire confirmed presence of neuropathic pain. After injection with 25 mg amniotic and umbilical cord particulate, the patient’s pain decreased from 7.3 before injection to 0.3 at 6 weeks postinjection. In addition, neuropathic symptoms significantly improved at 2 weeks and were not present by 6 weeks. By 63 weeks, two of the patients reported continued complete pain relief, while one patient underwent total knee replacement due to an allergy of a previously implanted unicondylar implant.

**Conclusions:**

This case series suggests that amniotic and umbilical cord particulate may be a viable alternative to reduce pain in patients with neuropathic pain.

## Introduction

The saphenous nerve is a sensory nerve that provides innervation from the medial thigh to the medial knee and great toe. A major branch of the saphenous is the infrapatellar nerve, which arises at the medial knee. This particular location and anatomy of this nerve makes it susceptible to damage from falls (contusion) and especially during knee surgery. In fact, iatrogenic injury to this branch has been documented since 1945 as a potential complication after medial arthrotomy [1]. Neuritis of the infrapatellar branch of the saphenous nerve (IPS) can also result from entrapment, bursitis, or patellar dislocation [2–5]. The pain can manifest on the medial aspect of the knee and radiate towards the tibial tuberosity area. The condition is relatively uncommon, accounting for <1% of adult patients with lower extremity pain [6]; however, this may be due to being underdiagnosed and having often unrecognized etiology of persistent anterior knee pain. In fact, incidence rates have been published as high as 22.2–65.7% of patients undergoing general arthroscopic knee surgery or anterior cruciate ligament (ACL) reconstruction, usually due to vertical skin incisions made perpendicular to the nerve anatomy [7–12]. Due to the increasing elderly population and number of total knee surgeries performed yearly, neuritis of the IPS will likely be seen more frequently.

Current conservative treatment for IPS neuritis is traditionally performed with nerve blocks containing steroid and local anesthetic [13, 14]. However, steroidal injections are associated with complications, including skin atrophy, and limitations such as short-term symptomatic relief that may require subsequent surgical intervention [3]. Cryoneurablation is another nonsurgical treatment that can provide symptomatic relief but with variable long-term outcomes [3]. Hence, there is a current unmet clinical need for treating IPS neuritis nonsurgically. Amniotic umbilical cord particulate is known to be antiinflammatory, antiscarring, and proregenerative and has been used in many orthopedic applications such as plantar fasciitis, facet joint syndrome, and knee osteoarthritis to reduce pain [15–20]. This case series highlights the use of amniotic umbilical cord particulate injection for the symptomatic improvement of recalcitrant neuritis of the IPS.

## Case presentation

Three patients with confirmed IPS neuritis were injected with 25 mg amniotic umbilical cord particulate (CLARIX FLO, Amniox, Miami, FL) mixed with 0.5 cc saline to the affected nerve under ultrasound guidance. Patient subjective evaluation of pain was reported preinjection, 2 weeks postinjection, and 6 weeks postinjection. The neuropathic pain diagnostic Douleur Neuropathique 4 questionnaire (DN4) and painDETECT questionnaire (PDQ) were also completed by the patients. The DN4 and PDQ are validated diagnostic tools to estimate the probability of neuropathic pain. A score of 4/10 in the DN4 is considered a positive test, whereas a score of 13 or above indicates possible neuropathy in the PDQ. All patients were treated at Orthopaedic Associates of St. Augustine, St. Augustine, FL 32086 by Beth Pearce, DPM.

Results showed mean pain numerical rating scale (NRS) score significantly improved from 7.3 at baseline to 1.7 at 2 weeks and 0.3 at 6 weeks (Tables 1, 2). By 63 weeks, two of the patients reported complete pain relief, while one patient underwent total knee replacement due to an allergy of an previously implanted unicondylar implant. No adverse events were noted directly related to the amniotic umbilical cord particulate.

**Table 1:** Pain scores for each subject for each time frame

Subject	Gender	Age	Time frame	Pain now	Strongest pain over 4 weeks	Average pain over 4 weeks	PDQ score	DN4 score
1	F	63	Baseline	7	8	8	13	4
			2 weeks	2	8	5	5	0
			6 weeks	0	2	2	0	0
2	F	61	Baseline	6	7	6	18	8
			2 weeks	3	6	0	13	1
			6 weeks	1	2	1	7	0
			63 weeks	0	0	0	4	0
3	F	55	Baseline	9	9	9	27	5
			2 weeks	0	9.5	4.5	13	1
			6 weeks	0	1	1	3	0
			63 weeks	0	1	1	4	0

Douleur Neuropathique 4 questionnaire (DN4); painDETECT questionnaire (PDQ)

**Table 2:** Average pain scores for all three subjects in each time frame

Time frame	Average pain now	Average strongest pain over 4 weeks	Average pain over 4 weeks	Average PDQ score	Average DN4 score
Baseline	7.3	8	7.7	19.3	5.7
2 weeks	1.7	7.8	3.2	10.3	0.7
6 weeks	0.3	1.7	1.3	3.3	0

### Case 1

The patient is a 63-year-old white female with a 10-year history of chronic right knee pain, for which she underwent multiple arthroscopies and a later unicondylar implant in 2012. Despite the interventions, her pain had failed to improve and has worsened thereafter. Consequently, she was transferred to Mayo Clinic’s Pain Management in Jacksonville, Florida, wherein she had good but only temporary pain resolution of no more than 2 weeks via nerve block intervention. Thus, she was diagnosed with infrapatellar saphenous neuralgia. She has described her pain as throbbing, aching, and often burning. At night, the pain was worse, causing an inability to sleep for “years,” which is classic to the diagnosis [21]. Standing and going up and down stairs exacerbated the symptoms that only marginally decreased even with multiple medications. During the 4 months prior to presentation, she reported the knee being very swollen and painful with flexion/extension, so a brace was utilized to limit movement.

**Fig. 1:**
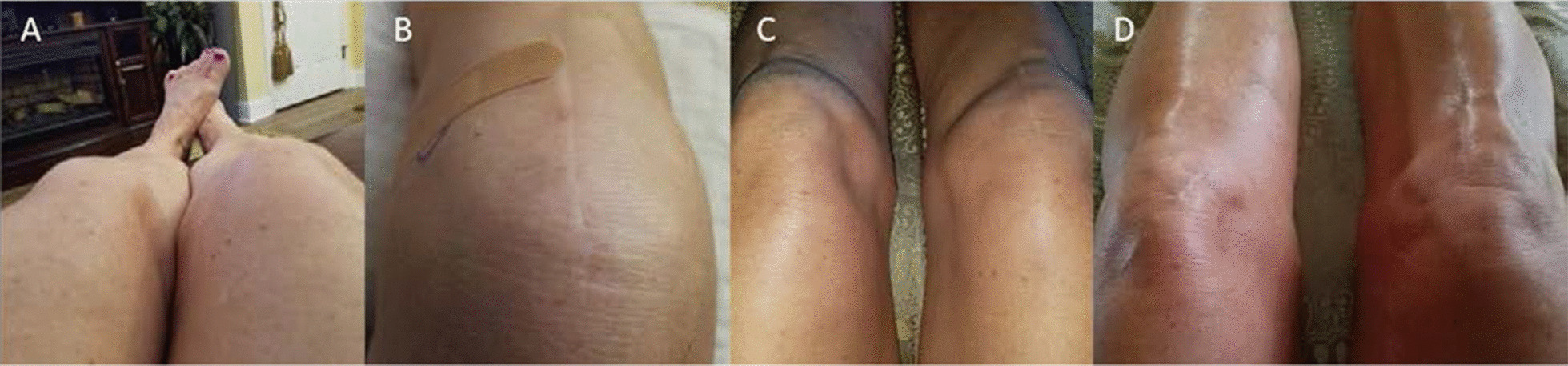
Clinical Presentation. Patient presented with chronic right knee pain and swelling (**a**). After amniotic umbilical cord particulate injection, complete symptomatic resolution was achieved within 3 days. The swelling had also reduced at 1 week (**b**), 1 month (**c**), and 6 weeks (**d**) post-injection and patient was able to regain complete ROM by 6 weeks

At presentation, the patient rated the pain in her right knee as 7/10 in severity on an 11-point numeric scale. On physical examination, there was a well-healed midline knee scar on the right extremity. She had mild/grade 2 medial joint laxity with 8 mm of anterior translation. There was accompanying tenderness with manipulation of the infrapatellar saphenous nerve region, as well as over the distal vastus lateralis and the iliotibial band. Edema at the level of the tibial fossa and hyperesthesia in the anterior aspect of the right leg/knee were noted (Fig. 1). Deep tendon reflexes were deferred as she had noted pain induced by the flexion and extension of the knee. She had no evidence of acute findings of infection or erythema, lymphangitis, or lymphadenopathy.

X-rays of the right knee showed good postoperative position of the prosthesis, suggesting no biomechanical reasons for the pain. Based on patient complaints, physical examination, and imaging data, the patient was diagnosed with right knee pain, infrapatellar saphenous neuralgia, and mild medial joint laxity.

The patient received 25 mg amniotic umbilical cord particulate, and her pain went away 3 days postinjection. She presented with 2/10 pain at 2 weeks and 0/10 pain at 6 weeks postinjection, with reduced swelling and regained range of motion (ROM). The pain remained subsided over a period of 10 weeks postinjection, however, then reoccurred. Further examination was performed, including a skin patch test for metal material that showed a high positive reach to nickel and cobalt. Therefore, the patient eventually underwent total knee replacement due to allergy to the initial partial implant.

### Case 2

A 61-year old white female with chronic (10–13 years) knee pain underwent left total knee arthroplasty in 2011 and right unicondylar arthroplasty in March 2016. Following the unicondylar implant, which was unremarkable, the patient had sudden onset of pain and stiffness. Thus, she was treated with several medications, including narcotics and acetaminophen, but reported no complete symptomatic relief. After 6 months, she was referred to our office where she presented with right knee pain rated 6/10 in severity. She described her pain as throbbing and aching that was intermittent, but sometimes caused complete loss of function in her knee. She noted her pain was aggravated while standing, flexing/extending (where sometimes she could not fully extend her knee), and especially going down stairs. Upon physical examination, there was a well-healed right midline knee scar. She had no evidence of acute findings of infection or erythema, lymphangitis, or lymphadenopathy. X-rays of the right knee identified excellent positioning of the arthroplasty without loosening, suggesting no biomechanical reasons for the pain.

To assess for potential infrapatellar saphenous neuralgia, a diagnostic IPS block with 2 mL Marcaine 0.25% plain and 0.5 mL of Depo-Medrol 40 was injected along the region of maximum pain, which was at the medial inferior portion of the knee. The patient tolerated the injection well and reevaluated her pain to be 0/10 but acknowledged it was only temporary relief. However, tenderness was noted distal to the block site, which suggested that the infrapatellar saphenous bifurcation from the saphenous nerve was somewhat more distal than initially suspected. Moreover, the patient reported more proximal pain following the course of the saphenous nerve, which indicated a “crush” that was addressed with a repeated IPS nerve block injection more proximal to the knee but without corticosteroid. A prescription for ½ tab of Desyrel nightly was also provided.

After 3 weeks, the patient developed progressive increase of pain in the right knee and rated the pain level as a 4/10 but sometimes an 8/10; however, she was not sure what exacerbated symptoms. On physical examination, she still felt pain while flexing and extending the knee, but was now able to perform a straight-leg raise. The remainder of the examination was unchanged from the previous consultation. To reconfirm diagnosis of infrapatellar saphenous neuralgia, an IPS block was repeated in the local area of the right knee region where there was maximum nerve tenderness. The patient tolerated the procedure well with a temporary reduction in pain and described the region as “numb.” Based on patient complaints, repeated diagnostic IPS blocks, and physical examination, the patient was diagnosed with infrapatellar saphenous neuralgia. Cryoablation, hydrodissection, and amniotic umbilical cord particulate treatment were discussed with the patient, and we asked her to return within 2 weeks. The patient then decided and received 25 mg amniotic umbilical cord particulate, and her pain decreased to 3/10 at 2 weeks and 1/10 at 6 weeks postinjection. The pain remained subsided over a period of 63 weeks postinjection.

### Case 3

The patient is a 55-year old overweight white female with a chief complaint of left foot/knee/leg pain for the previous month. She has an extensive history of left medial knee pain and has undergone meniscectomy and reports she is “bone-on-bone.” She rated her pain at 2/10 during the day, but at night 9/10 in severity, presumably due to standing all day as a nurse practitioner. She also reported hypersensitivity at night in the shower, especially with hot water. The patient has been taking acetaminophen, which provided some but not complete symptomatic relief. X-rays failed to reveal any fractures, dislocation, or soft-tissue lesions. Physical examination revealed normal range of motion with mild discomfort but normal strength. Tenderness was noted at the saphenous nerve distal to ankle, and symptoms were exacerbated with percussion along the nerve with manipulation of the IPSN. There were no worrisome lesions; however, multiple areas of venous varicosities were noted through both lower extremities, consistent with venous insufficiency. Based on examination, IPS neuralgia was diagnosed.

After consent was obtained, a diagnostic/ therapeutic IPS block with 2 mL Marcaine 0.25% plain and 0.5 mL of Depo-Medrol 40 was injected at knee region of maximal nerve tenderness. The patient tolerated the injection well and reported 100% pain relief and numbness. After 2 weeks, the patient developed progressive increase of pain received another diagnostic/therapeutic block of Marcaine/Depo-Medrol. Again, after 26 weeks, the patient returned with recurrent pain (subjectively rated 6 out of 10), and another diagnostic/ therapeutic block of Marcaine/Depo-Medrol was performed. At this point, alternative treatments such as cryoablation and amniotic therapy were discussed. Eight weeks later, the patient returned with recurrent pain and received 25 mg amniotic umbilical cord particulate, and her pain decreased to 0/10 at 2 weeks and 6 weeks postinjection. The pain remained subsided over a period of 63 weeks postinjection.

## Discussion

IPS neuritis is common after surgery as the nerve’s anatomical course makes it vulnerable during procedures such as harvesting hamstring or bone-patellar tendon-bone anterior cruciate ligament grafts, knee arthroscopy, high tibial osteotomy, arterial bypass, varicose vein surgery, femoropoliteal bypass, femoral artery thrombectomy, venous stripping surgery, tibial nailing, and knee replacement surgery, especially when longitudinal incisions are made [12]. Not surprisingly, the three cases presented herein had history of prior knee surgery and complained of pain characteristically neuropathic in nature with burning, tingling, and sensitivity [21]. At presentation, physical examination was performed to rule out instability and patellofemoral pain, radiographs showed no abnormality of bone and joints, and a positive Tinel sign was confirmed. Patients also did not exhibit central sensitization, which may account for failing to respond to traditional nerve blocks. Diagnosis was confirmed by local injection of anesthetic and steroid, which provided temporary relief for < 2 weeks.

Overall, the three patients treated with 25 mg amniotic umbilical cord particulate showed an improvement in chronic pain relief (from 7.3 at baseline to 0.3 at 6 weeks). These results are quite favorable compared with the literature. Previously, a retrospective study reported an 80% favorable outcome (defined as decrease in pain) at 4 months among 30 patients after two steroid nerve blocks of 0.25% bupivacaine and 25 mg triamcinolone diacetate [22]. In this study, the average pain level for all patients decreased from 6.4 ± 0.3 to 2.8 ± 0.5 (*p* < 0.001), which is comparable to our study in which the pain decreased from 7.3 to 0.3. However, our patients only received one injection, whereas the previous study had 1.9 ± 0.4 average injection blocks ranging from 1 to 5, with repeated nerve blocks every 3–4 weeks. Despite the success noted in these studies, there have been three studies that reported poor results with local injection. In one study, saphenous nerve entrapments in 19 patients ultimately required surgery due to no lasting pain improvement after local diagnostic injection [23, 24], although the follow-up time was not reported. In another study, only 12 of 32 patients (38%) reported success (although not defined) with nerve block therapy [25]. These conflicting results may be due to the use of only anesthetic, whereas the successful study also used corticosteroid as well. Yet Kopell injected two patients with hydrocortisone and only noted relief for 3–4 days [26]. Other potential nonoperative treatments for IPS neuritis include activity modification, physical therapy, transcutaneous electrical nerve stimulation, padding, oral or topical analgesics, and tricyclic antidepressants; however, no studies have been performed to determine their effectiveness. [13]

Due to limitations of the visual analog scale (VAS) measuring only general pain levels, and in order to differentiate between the different types of pain (that is, nociceptive and neuropathic pain), patients also completed DN4 and PDQ neuropathic pain questionnaires [27]. Nociceptive pain typically results from inflammation around the nerve, whereas neuropathic pain results from direct injury to the actual nerve. In our study, all three patients presented with present neuropathic pain before injection as determined by the questionnaires. After amniotic umbilical cord particulate injection, the patients’ subjective evaluation of neuropathic pain (as determined by DN4 and PDQ scores) decreased in coordination with the VAS pain levels. Despite the improvement in PDQ and DN4 scores being favorable, there are no published studies to compare these results for patients with IPS neuritis [28].

The findings from these three patients strongly suggest that the use of amniotic umbilical cord particulate is an effective nonsurgical option to provide improved pain relief and increased function in patients with IPS neuritis. The mechanisms of action might include the antiinflammatory and antiscarring compositional components of amniotic umbilical cord that collectively promote healing [29]. Amniotic umbilical cord is also known to contain neurotrophic factors that may aid in nerve regeneration and return of normal sensation [30]. These properties are advantageous as symptoms of anesthesia, hypoesthesia, hypesthesia, hyperalgesia, and allodynia stem from varying degrees of Wallerian degeneration [31]. Future prospective, controlled studies with longer-term follow-up and a larger patient population are warranted to confirm the use of this new treatment modality in neuritis.

## Conclusion

This case series highlights the utilization of conservative management with particulate amniotic umbilical cord to reduce chronic pain caused by neuritis of the infrapatellar branch of the saphenous nerve. Further investigation is required.

## Data Availability

All data generated or analyzed during this study are included in this published article.
